# Tips and Tricks to Improve Ergonomics, Efficacy, Versatility, and Overcome Limitations of Micro Percutaneous Nephrolithotomy

**DOI:** 10.3389/fsurg.2021.668928

**Published:** 2021-05-19

**Authors:** Sarvajit Biligere, Chin-Tiong Heng, Cecilia Cracco, Reshma Mangat, Chloe Shu-Hui Ong, Karthik Thandapani, Takaaki Inoue, Kemal Sarica, Ravindra B. Sabnis, Mahesh Desai, Cesare Scoffone, Vineet Gauhar

**Affiliations:** ^1^Division of Urology, Department of General Surgery, Ng Teng Fong General Hospital, National University Health System, Singapore, Singapore; ^2^Department of Urology, Cottolengo Hospital, Turin, Italy; ^3^Hara Genito-Urinary Hospital, Kobe University, Kobe, Japan; ^4^Department of Urology, Biruni University Medical School, Istanbul, Turkey; ^5^Muljhibhai Patel Urological Hospital, Nadiad, India

**Keywords:** PCNL, microperc, ECIRS, stone free rate, RIRS, retrograde intrarenal surgery

## Abstract

Percutaneous Nephrolithotomy (PCNL) has evolved over the decades from Standard to Mini to Ultramini PCNL to Micro-perc, with miniaturisation being the dominant theme and supine approach gaining momentum world over.

**Aim:** In literature, miniaturised PCNL with microperc needle access system has raised concerns of intrarenal pressure and has some limitations with its success for larger stones. Our tips and tricks explain how to overcome these pitfalls by utilising the full construct of the needle system to its maximum potential. These will in turn help make the procedure versatile, precise, ergonomical, and enhance a surgeon's experience with improved outcomes for patients especially in large renal stones.

**Materials and Methods:** We describe the limitations of microperc needle access as stated in literature and proposals by the co-authors using microperc for miniaturised access on how to overcome the same.

**Results:** A simplified table describing the limitations and tips and tricks on overcoming these is provided for quick reference.

**Conclusion:** As Technological advancements and techniques for miniaturised access in urolithiasis improve, we believe our suggestions will help surgeons overcome the quoted limitations of microperc needle access for miniaturised PCNL, making this a versatile, safe and efficacious technique even in large and complex stones. A multi centre trial will be the best way to validate the suggestions proposed in this article.

## Introduction

Global prevalence of nephrolithiasis has changed over the last few decades ranging from 7 to 13% in North America, 5 to 9% in Europe, and 1 to 5% in Asia. Management of stones can be expensive and has a high level of acute and chronic morbidity (1). The ideal stone surgery should achieve complete stone clearance in a single sitting with the least complications. For decades, PCNL has been the procedure of choice for large renal stone volume (2).

Larger and multiple percutaneous tracts provide good clearance, but have higher complication rates (3). Technical advancements have miniaturised PCNL with a singular goal to provide an easy and precise access into the collecting system, hence reducing the incidence of complications like bleeding, visceral injuries and nephron loss, which are attributed to bigger tract sizes (4). Mini-PCNL and Micro-perc (MP) needle access system are two major technical advancements that have evolved with an aim to avoid these complications (5).

**Figure 1:**
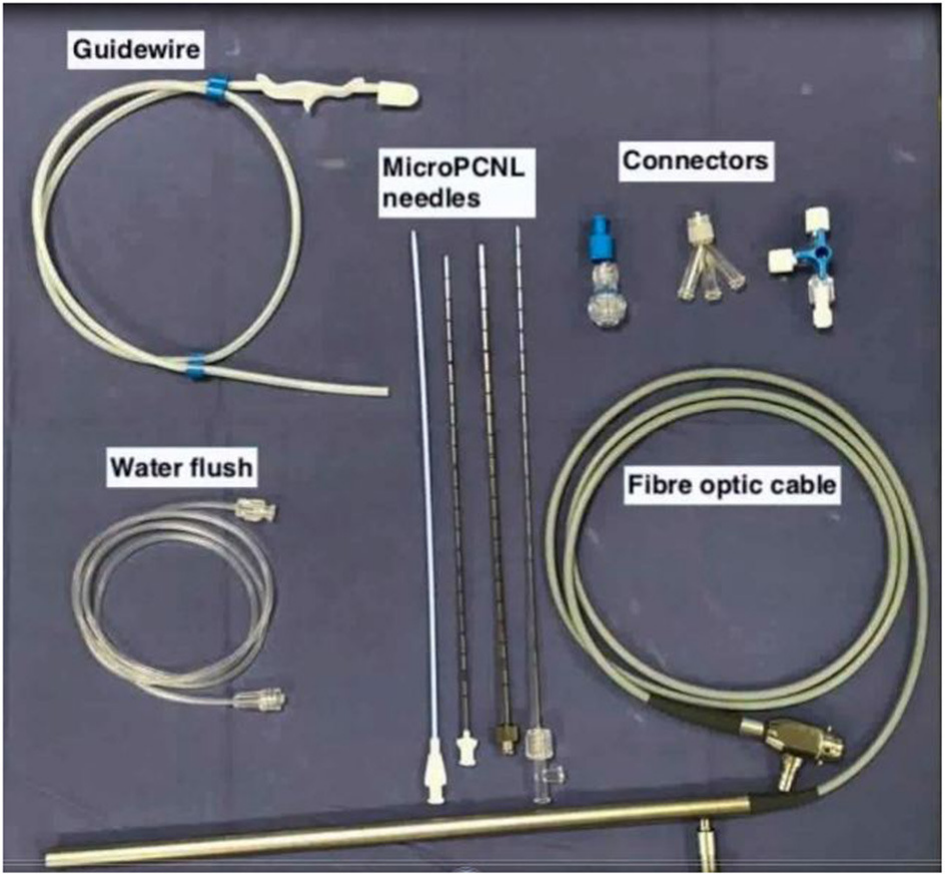
The Mini-micro perc set consisting of puncture needles, guidewire, and connectors.

**Figure 2:**
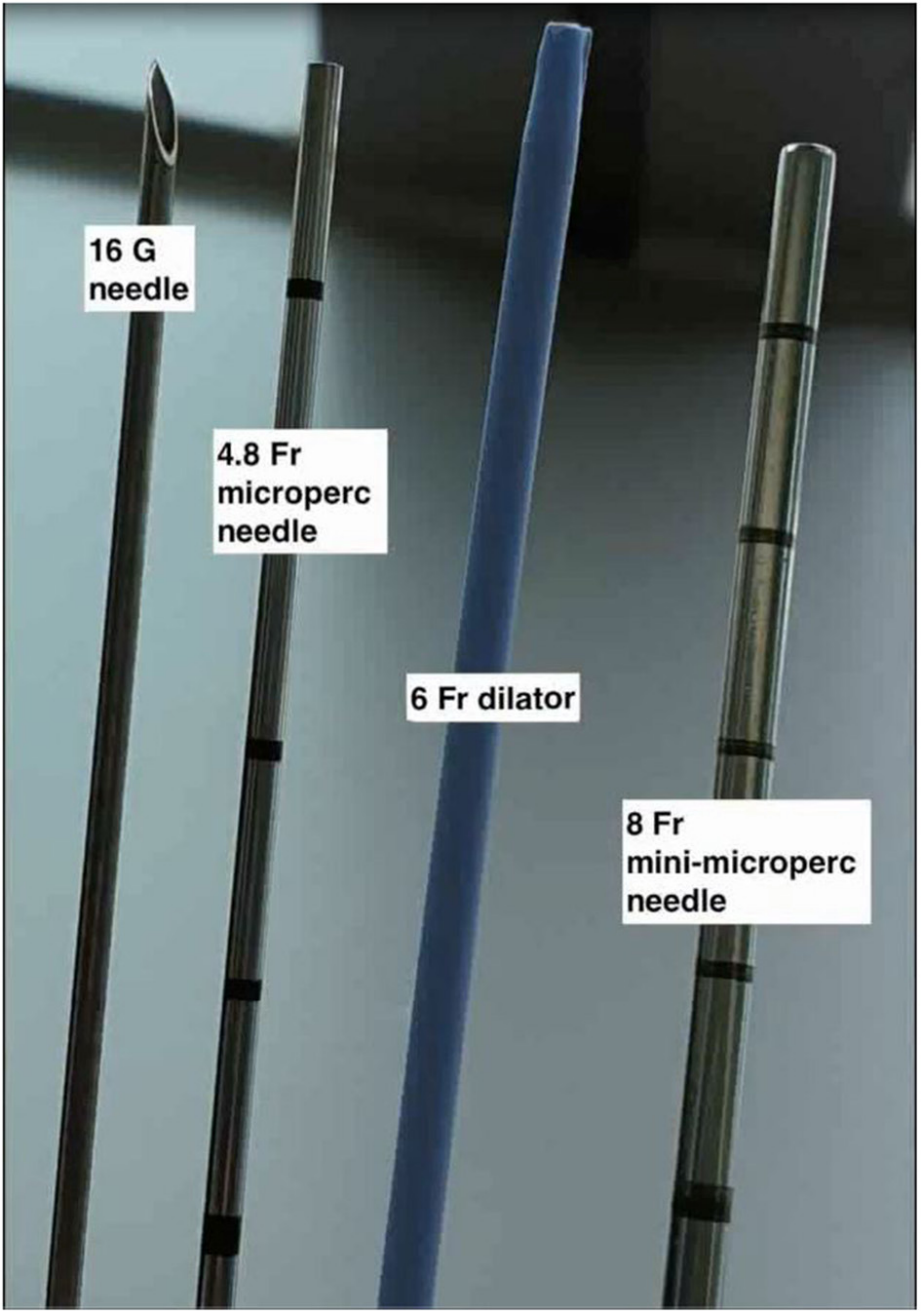
The micro and mini-microperc armamentarium.

PCNL by Micro-perc was described as a novel technique by Desai et al. (5) in 2011 wherein the procedure was completed via the 4.8Fr (16 Gauge) puncture needle. This was made possible by use of advanced and miniaturised optics, thus obviating the need for tract dilatation and its potential complications (4). The key components of a of Micro-perc system (shown in Figures 1, 2) include (6):

a) The needle: the 4.8Fr (16G) needle, has an outer sheath that acts as a conduit for passage of energy source or laser fibre, a central part comprising of a bevelled hollow needle, and an innermost part which is the radio-opaque stylet. In addition, there is also an 8Fr hollow sheath that can be used to “upsize” the tract, which is useful for tackling a larger stone burden. This was called the “Mini-Micro perc,” developed on suggestion by Sabnis et al. (6).
b) Optics: A flexible fibre optic telescope which consists of micro-optics 0.9 mm in diameter and 120 degree view with a 10,000 pixel resolution.
c) Energy source: For lithotripsy via the 4.8Fr microperc sheath, a 200 micron or smaller laser is the recommended choice. Alternatively, the 1.6 mm ultrasonic probe can also be used if the micro perc is converted to a mini-micro perc (8Fr) (6).
d) Assembly: The 4.8Fr Needle is a bevelled hollow sheath through which the optic fibre is inserted. The hub of the needle is compatible for attachment of a three way channel adaptor through which the laser fibre, optics, and irrigation are connected. The camera is fixed to the optic fibre cable and mounted on a central rod unlike conventional endourology systems where the camera is directly attached to the rod lens system (Supplementary Video 1).

## Technique

Renal access with a MP system is similar to a standard supine PCNL approach. This is performed with the 4.8Fr needle under fluoroscopic guidance either by bull's eye, biplanar, or triangulation method. This puncture can also be performed under ultrasound guidance. Access into the desired calyx can be aided by a retrogradely placed ureteric catheter providing calyceal dilatation (with saline for ultrasound-guided puncture or contrast medium for fluoroscopy-guided puncture) or under endoscopic guidance with retrograde access by a flexible ureteroscope.

## Limitations of MP Access

As described by Sabnis et al. (6), the major limitations of MP access on PCNL are:

1) Inability to retreive stone fragments due to the small tract size. This necessitates that the stone be dusted completely. Hence, this technique was proposed as best suited for stones upto 1.5 cm in size (6).
2) Possibility of generating high Intra-renal pressures (IRP) as it is a closed system with no fluid drainage vis a vis renal access sheath in PCNL.
3) Sepsis due to prolonged raised intrarenal procedure.

Based on our cumulative experience, we describe tips and techniques to overcome the above mentioned limitations and to make the MP access versatile and efficacious.

## Tips to Overcome Limitations of Micro PCNL

### Micro-ECIRS—MP PCNL + Endoscopic Combined Retrograde Intra Renal Surgery (ECIRS)

An advantage of using the MP system is that once the needle is in the Pelvicalyceal System (PCS), lithotripsy can begin instantly. The disadvantage being that, this is a closed system and hence only small stones can be tackled for fear of raised pressures, sepsis and inability to retrieve fragments.

**Figure 3:**
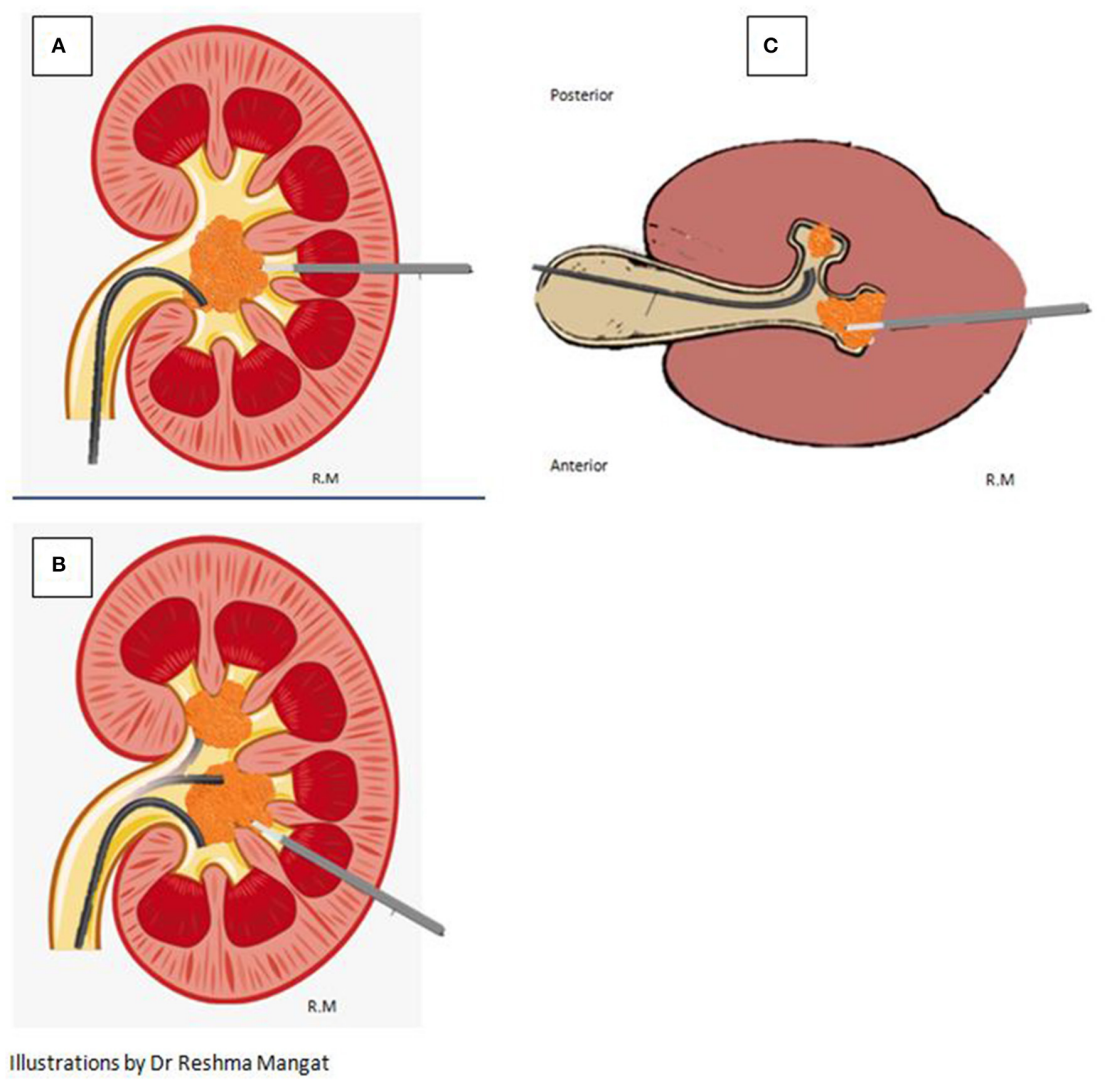
Versatility of the micro-perc approach resulting in efficacious lithotripsy is depicted. **(A)** Simultaneous Antegrade and Retrograde access to the same large stone. **(B)** Bi-directional simultaneous access to stones in different poles of the kidney. **(C)** Bi-directional simultaneous access to stones in different calyces in the same pole of a kidney.

However, when this procedure is combined with retrograde lithotripsy via the flexible ureteroscope placed via the ureteric access sheath (UAS) as a ECIRS, it allows for any stone to be dealt with simultaneously from antegrade and retrograde approach. Continuous fluid drainage via the UAS keeps vision clear and the IRP low. Fragments can be extracted or flushed out from the UAS. Additionally, stones in different locations can be accessed simultaneously (as depicted in Figure 3).

Apart from this benefit of ECIRS that has been elegantly summarised in literature by Cesare et al. (7) the position of the MP needle can be visually confirmed by the retrogradely placed flexible ureteroscope (FURS) guiding the needle precisely into the PCS avoiding any injury to collecting system (Supplementary Video 2).

Using miniaturised instruments like the MP needle and a small diameter FURS minimises the chance of instruments clashing and inadvertent damage during movement of both instruments in the PCS especially while using the laser.

### Applying the MATRIOSKA Technique

**Figure 4:**
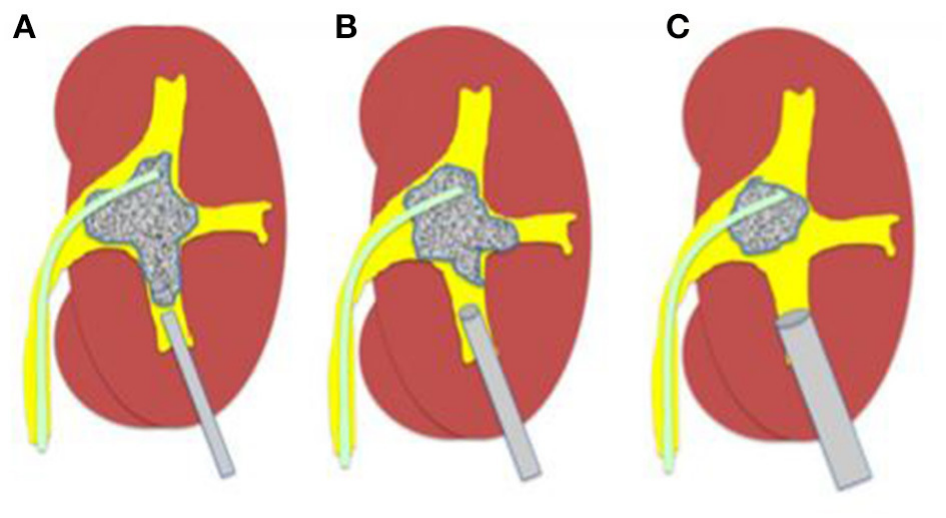
The Matrioska technique. **(A)** A microperc is utilised first to visualise the calyces system, insert quidewires, and to create space. **(B,C)** Once space has been created and vision improves, upsizing of the tract can be done to a micro-mini or mini sheath for adequate lithotripsy according to the energy source available.

MP PCNL is cited as inefficient for large stones due to small access (6). This is overcome by using the Matrioska technique decribed by Zanetti et al. (8) whereby the 4.8Fr puncture can be upsized to 8 or 12Fr sheath or even larger tailoring access according to patient anatomy and stone size. This makes the procedure more flexible and allows better drainage with “vacuum cleaner effect” for retrieval of fragments. By step wise progression, the surgeon has full flexibility to go from small to large in a single dilatation unlike conventional mini PCNL (as depicted in Figure 4).

**Figure 5:**
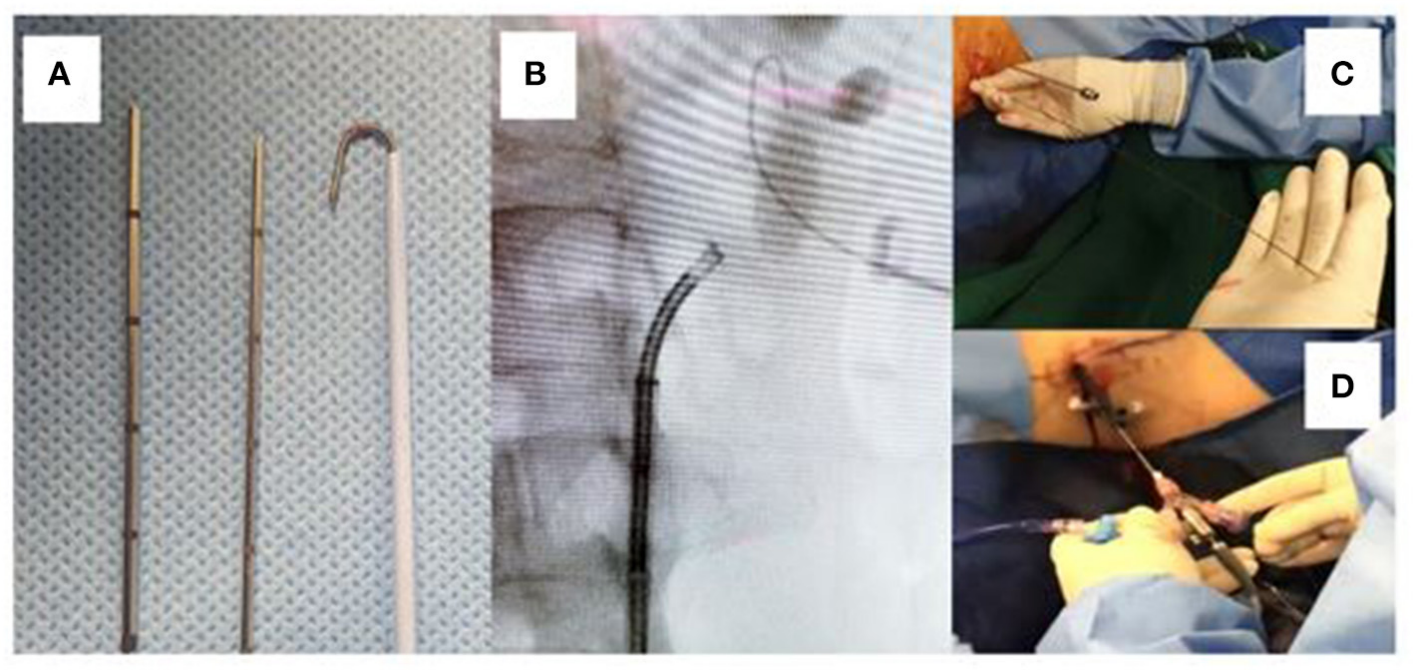
**(A)** The micro and micro-mini needles with a J-tip super-stiff wire. **(B)** Fluoroscopic image showing the J-tip wire beyond the stone, securing access. **(C)** The super stiff wire remains in line with the puncture and perpendicular to the patient making dilatation and upsizing easy. **(D)** A larger calibre sheath is used here to upsize the tract, while the 4.5 Fr micro needle remains as a “nephroscope” for effective lithotripsy.

The robust super-stiff, short-length, angled-tip guidewire included in the MP set makes the upsizing of the tract easy and effective (as depicted in Figure 5).

### Lowering Intrarenal Pressures

High IRP is deterimental for ureteroscopy and PCNL (9). Renal pelvic pressures (>30 mmHg) promote pyelovenous and pyelolymphatic reflux leading to infectious complications, systemic inflammatory response syndrome (SIRS), and sepsis leading to prolonged hospital stay (10–15).

Concerns of higher IRPs in a MP PCNL can be mitigated by placing a UAS for RIRS (16–19) with or without suction and or using a small diameter (10–12Fr) percutaneous renal access sheath.

As there is adequate space between the needle and sheath, it promotes faster stone clearance of fragments and dust by combining vaccum cleaner effect, active flushing and if available, by application of direct suction to the renal access sheath to aspirate all the dust whilst uniformly maintaining low IRP.

### Harnessing the Power of Newer Lasers as Energy Source in Lithotripsy

Newer and more powerful high-power Ho-YAG systems combined with improved techniques such as popcorning, popdusting, and high-speed dusting using Moses technology ensure finer fragments shortened lasing time and complete stone clearance (20).

Thulium Fibre Laser (TFL) has been shown to be more efficacious with certain distinct advantages as described by Kronenberg and Traxer (21). Firstly, TFL is smaller than a Holmium:YAG laser fibre. Secondly, it creates smaller sized fragments and more dust particles when compared to the Holmium laser.

Alternatively, by upsizing to a Micro-mini perc, a 1.6 mm ultrasonic lithotripter can break and evacuate the fragments easily (6). Tiny fragments the size of a laser fibre can be flushed out easily through the mini-micro PCNL sheath and also via the UAS in Micro-ECIRS (Supplementary Video 4).

## Tips to Make the Puncture More Precise

### Direct Visual Access (Visual Fascial Access Single-Step Technique) (V-FAST)

**Figure 6:**
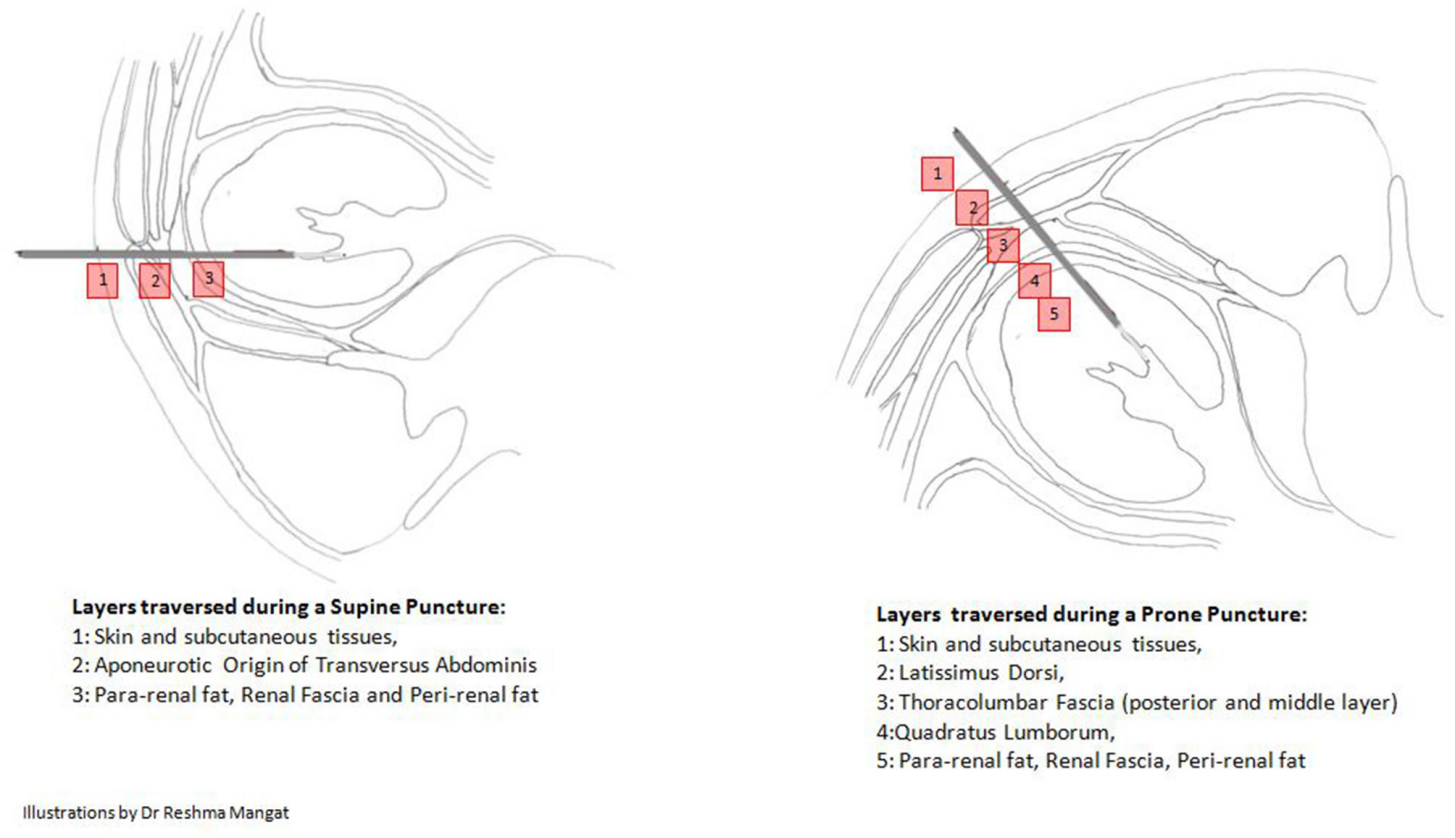
The different fascias traversed depending on the approach of puncture.

The 4.8Fr needle when connected to the fibre optic cable becomes the “All seeing needle” (5). This allows entry under direct vision and recognition of the different fascial layers from the skin incision to the pelvicalyceal system (PCS) (as depicted in Figure 6). During this step, any mild bleeding that hinders vision can be cleared with gentle and short bursts of water irrigation with a 5cc syringe. This ensures an easy, single step, direct visual, precise, and proper tract creation with visualised entry into the PCS, avoiding any major blood vessels, akin to using a visual optical trocar in laparoscopic surgery (22). The absence of an amplatz sheath significantly reduces complications associated with bleeding and loss of renal parenchyma (23).

The precision of V-FAST prevents under or over dilation and inadvertent injury or perforation of the renal pelvis. Entry into the desired calyx is ensured under Fluoroscopic or Ultrasound guidance.

### Ultrasound-Assisted Puncture: Wide Band Doppler for Delineation of Arterial Anatomy

It has been well-established that percutaneous renal access with ultrasound-assisted puncture improves outcomes and minimises complications by avoiding visceral injury, intra-renal vascular injury, and minimising radiation exposure. It allows a straight and short access to the collecting system with minimal morbidity (24–26).

Takaaki et al. (27) have described the benefit of using wide band doppler in ultrasound-guided puncture for Mini-ECIRS. They have shown that when ultrasound is coupled with B-mode doppler (wide band), blood vessels traversing the renal parenchyma can be effectively identified in real time and avoided.

The 4.8Fr needle has good acoustic properties and can be easily visualised during an ultrasound-guided puncture. Micro-perc access, combined with the above described principle of wide band doppler allows for precise entry into the PCS and significantly reduces haemorrhagic complications. This makes the micro-perc puncture precise, versatile, and effective (Supplementary Video 3).

## Benefitting From the Ergonomics of a Light Weight MP System

All the components of the MP system provide excellent ergonomics to the surgeon during and after gaining access into the PCS in the following ways:

1) The optic fibre cable and the camera assembly—As the camera system stack is off-table, the needle is completely free from the weight of the camera system. This makes handling and manoeuvring the needle and shaft easier and more convenient for lithotripsy, which is especially useful in tackling larger stone volumes for longer operative periods.
2) The needle and sheath are very light and can be easily manoeuvred into all calyces. Its 15 cm length makes it a useful tool especially in obese individuals with higher skin-to-stone distances.
3) Micro-ECIRS with MP system—The entire procedure in a supine access and with a light weight disposable flexible ureteroscope can be performed with both surgeons seated throughout. This significantly improves the ergonomics of the operation, reduces muscle fatigue, and potentially improves surgeon performance (28, 29).

## Conclusion

Supine PCNL with MP access is a versatile procedure that can be modified and optimised to overcome literature cited limitations, hence, personalising the management of stones for each patient. This procedure has clear advantages in terms of reduced hospital stay, lower complication rates and high SFR as evidenced by numerous randomised studies (6).

Micro-ECIRS can serve as a natural progression of PCNL by MP system. These modifications are beneficial in paediatric population, anomalous kidneys, patients with physical deformities, or on anti-coagulation.

Our experience-based tips and tricks to overcome the limitations and maximise the potential of MP access are summarised in Table 1.

**Table 1:** Salient advantages of PCNL by MP system and suggestions to overcome its limitations.

**Tips to overcome limitations of PCNL by MP access system**	**Limitations as cited in literature that can be overcome** A) Inappropriate for large stone B) Raised intrarenal pressure C) Laser lithotrispy is inefficient D) Fragments cannot be retrieved
(1) Micro-ECIRS (30)	a) Bi-directional access to staghorn calculi b) Bi-directional access to stones in different locations c) Complex anatomy stones, patients with physical deformities d) Reduce IRP with UAS e) Active Flushing of stone fragments for retrieval f) Aspiration of dust if a suction UAS is used
(2) Matrioska technique	a) Upsizing of tract—personalised stone approach b) Vacuum cleaner effect is possible when combined with a nephrostomy sheath for fragment retrieval
(3) Lasing techniques	a) High power lasers like TFL/Moses increase dust, smaller fragments b) Faster fragmentation; shorter intraop times c) Larger/multiple stones can also be tackled
(4) Intra-renal pressures	a) Raised IRP can be mitigated by using UAS or suction percutaneous renal access sheaths which converts a closed MP system into a continuous drainage system b) Decreased IRP mitigates risk of sepsis when larger/multiple or harder stones are tackled
(5) Improved ergonomics of a light weight MP system	a) Off-table camera system Light weight, easy to handle, and pen like grip allows for easy strain free manipulation in the PCS b) Combination with supine micro-ECIRS using a light FURS minimises neck strain and fatigue for both the seated surgeons
**Improving MP access precision**	**Potential advantages**
(1) Needle design	a) Better acoustic property for US puncture b) Better manouvreability with a pen like grip due to its light weight c) Very useful in obese patients (15 cm length) d) Only system to allow a V-FAST puncture for PCNL
(2) Wide band Doppler	a) Better delineation of vascular anatomy during access

## Author Contributions

SB contributed to design of the paper, the first and corresponding author and contributed to critical revisions of the draught along with assimilation and editing of images and videos, and also wrote the first draught of the paper. VG is the last author and contributed toward concept and design of paper and critical revision. CS, CC, KS, RS, MD, and C-TH contributed toward critical revision of the paper. TI contributed toward creation of a video and critical revision. RM contributed as a creative editor by creating images. CO and KT also contributed toward formatting of the draught and creative content and assimilation of data. All authors contributed to the article and approved the submitted version.

## Conflict of Interest

The authors declare that the research was conducted in the absence of any commercial or financial relationships that could be construed as a potential conflict of interest.

## Supplementary Material

Supplementary Video 1 Assembly of the system.

Supplementary Video 2 Retrograde guided access into PCS.

Supplementary Video 3 US wide band doppler ECIRS puncture.

Supplementary Video 4 Antegrade Lithotripsy with flushing out of fragments from UAS.
